# Reverse Transcriptase-Coupled Quantitative Real Time PCR Analysis of Cell-Free Transcription on the Chromatin-Assembled *p21* Promoter

**DOI:** 10.1371/journal.pone.0023617

**Published:** 2011-08-23

**Authors:** Jeong Hyeon Park, Natisha Magan

**Affiliations:** Institute of Molecular BioSciences, Massey University, Palmerston North, New Zealand; University of Leeds, United Kingdom

## Abstract

**Background:**

Cell-free eukaryotic transcription assays have contributed tremendously to the current understanding of the molecular mechanisms that govern transcription at eukaryotic promoters. Currently, the conventional G-less cassette transcription assay is one of the simplest and fastest methods for measuring transcription in vitro. This method requires several components, including the radioisotope labelling of RNA product during the transcription reaction followed by visualization of transcripts using autoradiography.

**Methodology/Principal Findings:**

To further simplify and expedite the conventional G-less cassette transcription assay, we have developed a method to incorporate a reverse transcriptase-coupled quantitative real time PCR (RT-qPCR). By using DNA template depletion steps that include DNA template immobilization, Trizol extraction and DNase I treatment, we have successfully enriched *p21* promoter-driven transcripts over DNA templates. The quantification results of RNA transcripts using the RT-qPCR assay were comparable to the results of the conventional G-less cassette transcription assay both in naked DNA and chromatin-assembled templates.

**Conclusions:**

We first report a proof-of-concept demonstration that incorporating RT-qPCR in cell-free transcription assays can be a simpler and faster alternative method to the conventional radioisotope-mediated transcription assays. This method will be useful for developing high throughput in vitro transcription assays and provide quantitative data for RNA transcripts generated in a defined cell-free transcription reaction.

## Introduction

The p53 tumor suppressor has been extensively studied for its role as a transcriptional activator for a range of downstream target genes [1]. The *p21* gene is one of the most studied direct downstream target genes of p53, which is inducibly activated through two consensus p53 response elements located at −2.3 kb and −1.4 kb [2]. This p53-dependent *p21* gene activation has also been shown to require the recruitment of p300 histone acetyltransferase (HAT) and localized nucleosome acetylation which in turn facilitates transcription through chromatin modifications [3]. Since the laboratory of Robert Roeder introduced cell-free transcription of RNA polymerase II in a test tube [4], detailed *in vitro* studies of the transcription machineries have illuminated many critical molecular mechanisms for eukaryotic transcription [5]. Cell free transcription assays are an extremely powerful tool to dissect or recapitulate the functional roles of diverse transcription factors and cofactors by setting up biochemically defined transcription reactions in a test tube. The G-less cassette *in vitro* transcription assay was developed to speed up the measurement of RNA polymerase II-dependent transcripts on DNA templates [6]. This method generates radiolabelled G-less RNA products of a defined length which are then visualized using polyacrylamide gel electrophoresis and autoradiography. However, the G-less cassette transcription assay is still a time and labour-consuming procedure, undertaking multiple technical steps that commonly require two days and more [7]. *In vitro* transcription for class II promoters is also known to be inefficient in terms of template usage [8]. In a conventional G-less cassette transcription assay, typically 50 to 100 ng of the template DNA is required in the transcription reaction along with ATP, CTP and [α-^32^P]UTP supplemented with unlabelled UTP. Considering the limitations of inefficient template usage (0.03 transcripts synthesized per strong promoter-containing DNA template [8]) along with a low rate of radioactive UTP incorporation into a RNA transcript (approximately one in 8 transcripts is radioactively labelled [7]), detection would only be possible if over hundred picogram amounts of the RNA transcript was produced via the G-less cassette transcription assay. In order to bypass the radioisotope labelling of RNA and time-consuming steps of electrophoresis and autoradiography, a RT-qPCR method was first employed in this study to measure RNA levels in the transcription reaction. To this end, additional steps were also incorporated to remove template DNA to a reasonable level such that it does not interfere with the final quantitative analysis of RNA product from a transcription reaction.

Here we outline a method for a radioisotope-free *in vitro* transcription assay by using biotin-labelled template DNA, standard RNA extraction techniques and RT-qPCR assay. We have successfully measured *p21* promoter-driven transcripts from a model system of p53 and p300-dependent chromatin transcription and demonstrated the validity of our method by comparing the results to a conventional G-less cassette transcription assay.

## Results and Discussion

### Generation of *p21* promoter-driven G-less cassette

**Figure 1:**
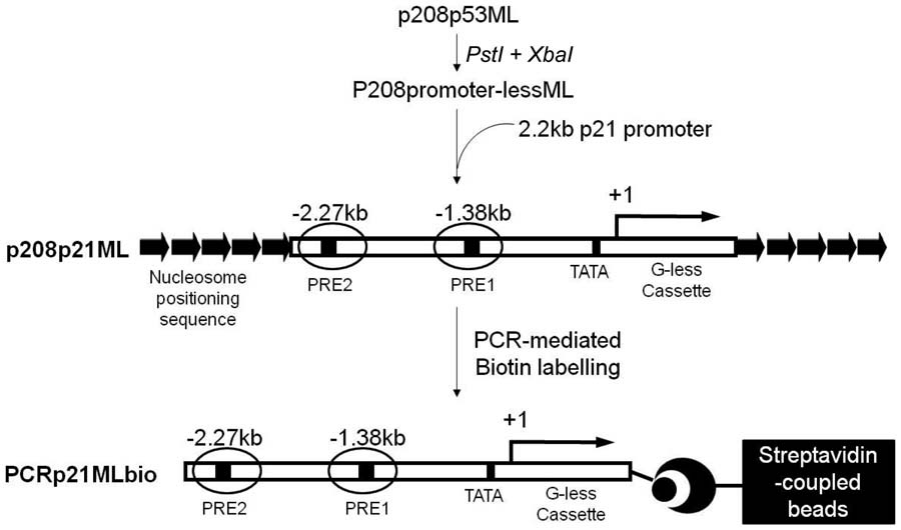
Schematic presentation of the *p21* promoter template DNA. Plasmid p208p21ML (derived from p208p53ML and pWWP-luc) was used as a template in the conventional G-less cassette *in vitro* transcription assay. Biotinylated linear template DNA (PCRp21MLbio), amplified from p208p21ML was used as naked DNA and chromatin-assembled templates in the radioisotope-free *in vitro* transcription assay. Transcription start site is indicated by +1. PRE: p53 response element, TATA: TATA box.

To assess the feasibility of RT-qPCR to replace the radioisotope labelling in the transcription assay, we used a natural *p21* promoter that has been shown to be activated by p53- and p300-dependent manner on the chromatin template *in vitro* [3]. To construct the *p21* promoter-driven G-less cassette (Figure 1), the promoter from p208p53ML [9] plasmid was removed by PstI and XbaI restriction enzyme digestion and replaced with a PCR-amplified *p21* promoter derived from a pWWP-luc plasmid [10] using primer set, p21PCRpWWP (table 1). The resultant construct, p208p21ML contained the *p21* promoter-driven G-less cassette surrounded by 5S rRNA nucleosome positioning sequences and was used for the conventional G-less cassette transcription assay in this study. In order to be able to remove template DNA/chromatin after an *in vitro* transcription reaction, we generated a biotinylated linear DNA (PCRp21MLbio) of the *p21* promoter template using primer set, Biop21PCR (table 1). PCRp21MLbio was used for as the naked DNA template or chromatin-assembled template for our revised cell-free transcription assay. Streptavidin-coupled beads were later used to immobilize biotin-labelled template, which provided an essential DNA template depletion step during the procedure. Assuming less than one picogram of contaminating template DNA is acceptable in the final qPCR analysis, the DNA template depletion step is required to remove 99.999% of the initial input DNA template (1 pg out of 100 ng template DNA). We found that conventional RNA purification steps in conjunction with DNase I treatment cannot sufficiently remove contaminating DNA from the transcription reaction (data not shown). In order to obtain a desirable amount of DNA template depletion, we used biotin-labelled template DNA which was immobilised using streptavidin-coupled beads in the transcription reaction and facilitated the removal of template DNA after the transcription reaction.

**Table 1:** Sequences of PCR primer pairs.

p21PCRpWWP	5′ gcttggcctgcaggctgtggctctgattggct	3′ ccgccttctagaggcgacccgcgctcggccca
Biop21PCR	5′ ctgcaggctgtggctctgattggct	3′ Biotin-gagtggaatgagaaatgagtgtgag
p21RT-PCRbio	5′ ttttatgattggggataagattgaa	3′ cctttccatatcccctccac

In a conventional transcription assay, the G-less cassette must be cloned at the precise start site of transcription and results in a chimeric fusion of an artificial G-less cassette and a natural promoter. However, if downstream promoter elements play a significant role in regulating transcription activity, the G-less cassette containing promoter may not recapitulate actual physiological regulation of the transcription [11]. This disadvantage may be overcome using the RT-qPCR method since the sequence downstream of promoter is not restricted to a G-less sequence and therefore can be easily modified to a natural promoter and coding sequence.

### Preparation of proteins for transcription reaction on chromatin template

**Figure 2:**
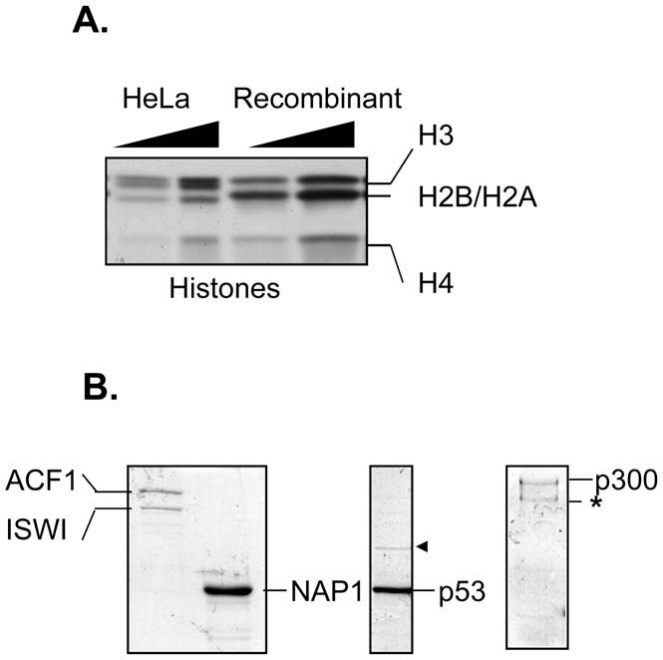
Analysis of recombinant histones, chromatin assembly factors, transcriptional activator, and chromatin modifier. (A) Xenopus recombinant histones (1 and 2 µg) were resolved in 12% SDS-PAGE and stained with Coomassie Brilliant Blue R-250. (B) Copurified drosophila Flag-ACF1 and ISWI, Histidine-tagged NAP1, transcriptional activator Flag-p53, and chromatin modifier Flag-p300 are shown with Coomassie Brilliant Blue R-250 staining. ◂, copurified protein from *E. coli*. *, a partially degraded p300.

Detailed methods on the preparation of recombinant factors for chromatin assembly and transcription reaction have been previously described [12], [13]. HeLa nuclear extracts were used as a source of general transcription machinery [8]. Recombinant Xenopus histones were individually expressed in *E. coli* and purified as previously described [12]. Reconstituted histone octamers were purified using gel filtration chromatography and compared with HeLa core histones (Figure 2A). Histidine-tagged NAP1 and Flag-tagged p53 were expressed in *E. coli* and purified using Ni-NTA resin and M2 agarose, respectively (Figure 2B) [12]. ACF Chromatin assembly factor (ACF1+ISWI) and p300 histone acetyltransferase were expressed in Sf9 cells and purified using M2 agarose [13]. All protein preparations used in this study were examined using SDS-polyacrylamide gel electrophoresis (SDS-PAGE) and Coomassie Blue R-250 staining (Figure 2) and the relative concentrations were determined using bovine serum albumin as a standard. The homogeneity of preparations was comparable to similar preparations used in other studies [14].

### In vitro chromatin assembly of p208p21ML plasmid and synergistic transcriptional activation by p53 and p300

The *p21* promoter was modified in the p208p21ML plasmid with the fusion of G-less cassette and therefore its transcriptional regulation could be different from *p21* promoter driven-luciferase plasmid used in the previous studies [3]. Before attempting real time PCR analysis of *in vitro* transcription assay, we examined if p53 and p300-dependent transcriptional activation in p208p21ML was comparable to that in previous studies [3].

**Figure 3:**
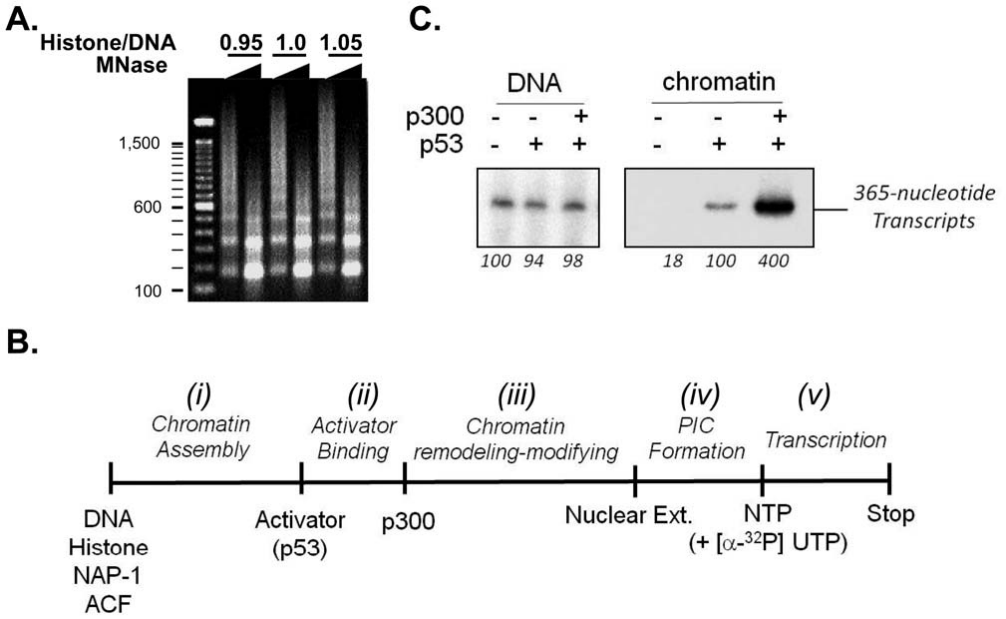
Conventional G-less cassette transcription assay. (A) Micrococcal nuclease (MNase) digestion of chromatin-assembled p208p21ML plasmid. 500 ng of assembled chromatin was digested with MNase and resolved using 1.2% agarose gel electrophoresis and ethidium bromide staining. The mass ratios of core histones to DNA are indicated. 100 bp DNA ladders were used as a size marker. (B) Summary of the *in vitro* transcription reaction is outlined (left to right) from chromatin assembly to the transcription reaction involving [α-^32^P] UTP-mediated labelling. PIC, preinitiation complex. (C) p53- and p300-dependent transcription of naked (left panel) and chromatin-assembled (right panel) p208p21ML plasmid. G-less cassette transcript of 365 bp was resolved in the 8 M urea-PAGE gel and visualized by autoradiography. Relative signal intensities were quantified using phosphoimager software and values are indicated at the bottom of the autoradiogram.

To examine *p21* promoter activity of the p208p21ML plasmid in a conventional G-less cassette transcription assay [7], chromatin assembly was performed as previously described [12] and the quality of the reconstituted chromatin was assessed by micrococcal nuclease digestion [15] (Figure 3A). The characteristic DNA ladders that were observed after MNase digestion demonstrates that the *in vitro* assembled chromatin mimics physiologically spaced nucleosomes and therefore deemed suitable for a cell-free transcription assay.

Conventional G-less cassette transcription assays were performed as described previously [13] and summarised in Figure 3B (ii to v). Briefly, 50 ng of supercoiled plasmid DNA template or chromatin was first incubated with 10 ng of purified p53 in a HAT reaction buffer (20 mM Tris-HCl; pH 7.6, 50 mM KCl, 5 mM DTT, 10 mM sodium butyrate, 2 mM MgCl_2_, 5% glycerol) at 30°C for 20 min. Then, targeted nucleosome acetylation was performed in the HAT reaction by adding 15 ng of purified p300 and 2 µM acetyl-CoA followed by an incubation at 30°C for 30 min. To allow for the assembly of the preinitiation complex on the promoter template, 27 µl of a transcription reaction mixture (6 µl of transcription buffer (200 mM HEPES; pH 7.9, 40 mM MgCl_2_), 0.48 µl of 1 M DTT, 1.22 µl of BSA (10 mg/ml), 0.3 µl of RNasin (10 U/µl), 6 µl of BC150 buffer (20 mM Tris-HCl; pH 7.9, 0.2 mM EDTA, 150 mM KCl, 20% glycerol) and 10 µl HeLa nuclear extracts (10 mg/ml)) was added to 30 µl of the HAT reaction and incubated for 20 min at room temperature. The transcription reaction was now initiated by adding 3 µl of nucleotide substrates (12 mM ATP, 12 mM CTP, 0.5 mM UTP and 2 mM 3′-O-methyl-GTP) and 12.5 µCi (10 µCi/µl) of [α-^32^P]UTP (3,000 Ci/mmol)) and incubated for 50 min at 30°C. After treatment with 10 U of RNase T1 for 15 min, the radioisotope-labelled RNA transcript was purified using multiple steps including proteinase K digestion, phenol/chloroform extraction and ethanol precipitation. The final RNA pellet was resuspended in 10 µl of formamide loading buffer (98% Formamide, 0.5 mM EDTA, 0.1% xylene cyanol, 0.1% bromophenol blue) and analysed by electrophoresis using a 5% polyacrylamide-8 M urea gel. The final result of ^32^P-labelled RNA transcript was visualised by exposure to X-ray film (Figure 3C). The results demonstrate that the naked DNA template of p208p21ML produced high basal levels of transcription which was not affected by either p53 or p300 (Figure 3C, left panel). However this high basal level of transcription was significantly repressed when using the chromatin-assembled *p21* promoter (Figure 3C, right panel). Only a modest enhancement of transcription was observed with p53 alone on the chromatin template, which, however, was significantly stimulated by co-incubating with p300 histone acetyltransferase. This result suggests that main function of p53 in activating *p21* promoter could be mediated through recruiting chromatin modifying /remodelling activity to the promoter to relieve chromatin-mediated repression.

Previous studies using *p21* promoter-luciferase plasmid (pWWP-luc) and primer extension analysis [3] demonstrates that transcriptional activation of chromatin-assembled *p21* promoter requires p53 in conjunction with p300 histone acetyltransferase whereas naked DNA template produces a high level of basal transcription regardless of the presence of p53 and p300. Our results are in line with this earlier study and indicate that the p208p21ML plasmid, like the pWWP-luc plasmid, can be successfully used as a template for cell-free transcription analysis of *p21* promoter.

### Real time PCR analysis of in vitro transcription assay from a naked DNA template

**Figure 4:**
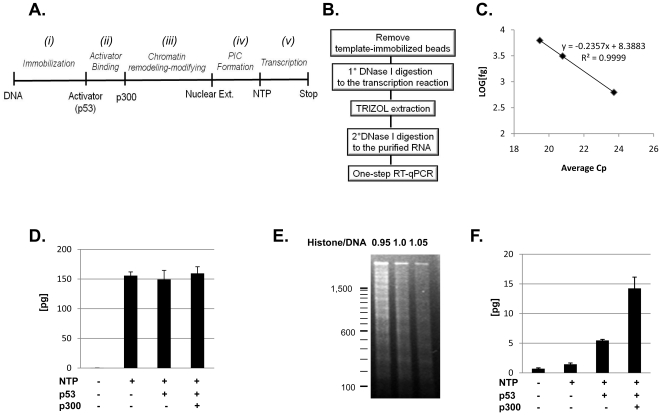
Real time PCR analysis of *in vitro* transcription assay from naked DNA and chromatin templates. (A) Summary of the *in vitro* transcription reaction is outlined (left to right) from a template immobilization on the streptavidin-coupled agarose to the radioisotope-free transcription reaction. (B) Schematic presentation of the procedures that are followed after the transcription reaction. (C) Standard curve of biotinylated template DNA (625/3,125/6,250 femtogram) from a RT-qPCR analysis. (D) Transcription output of naked *p21* promoter-driven G-less transcript. Triplicate samples were analyzed by a RT-qPCR and absolute quantification was performed based on the standard curve in panel (C). The standard deviation is indicated by error bars. (E) MNase digestion of chromatin-assembled biotinylated *p21* promoter template (PCRp21MLbio). 500 ng of assembled chromatin was digested with MNase and resolved using 1.2% agarose gel electrophoresis and ethidium bromide staining. The mass ratios of core histones to DNA are indicated. (F) Transcription output of chromatin-assembled *p21* promoter-driven G-less transcript. Triplicate samples were analyzed by a RT-qPCR and absolute quantification was performed based on the standard curve. The standard deviation is indicated by error bars.

A summary of the transcription procedure using RT-qPCR analysis is outlined in Figure 4A. The procedure is similar to the conventional G-less cassette transcription procedure (figure 3B) except for the template immobilization step (i), transcription reaction (v) and subsequent follow up procedures that are outlined in Figure 4B. Streptavidin-coupled agarose was preblocked with preincubation buffer (20 mM HEPES; pH 7.5, 200 mM NaCl, 1 mM EDTA, 10% glycerol, 5 mg BSA/ml and 0.1% NP-40). A total of 50 µl of the 50% streptavidin-coupled agarose suspension was incubated with 1 µg of PCRp21MLbio DNA in 500 µl of binding buffer (20 mM HEPES; pH 7.5, 2 M NaCl, 1 mM EDTA, and 10% glycerol) at room temperature for 1 hr with constant agitation. The efficiency of template DNA immobilization was calculated by measuring the amount of unbound DNA and was generally 50% or less than the amount of input DNA. The PCRp21MLbio-immobilized resin was washed thoroughly with HEG 50 buffer (25 mM HEPES; pH 7.6, 50 mM KCl, 0.1 mM EDTA, 10% glycerol) and resuspended in the same buffer to generate a final concentration of 16 ng PCRp21MLbio DNA/µl. The activator binding, chromatin remodelling-modifying and PIC formation steps were carried out as described in the conventional G-less transcription assay, followed by a radioisotope-less transcription. Briefly, a total of 3 µl of 20× nucleotide mixture (12 mM ATP, 12 mM CTP, 12 mM UTP and 2 mM 3′-O-methyl-GTP) was added to initiate transcription. After incubating the transcription reaction at 30°C for 50 min with constant agitation, the reaction mixture was centrifuged at 3,000 g for 1 min and the pellet of the template-immobilized resin was discarded. The supernatant was further treated with 10 U of RNaseT1 and 20 U of DNase I at 37°C for 20 min. After adding 10 µg of glycogen as a carrier, the reaction mixture was extracted using 200 µl of TRIZOL LS and 40 µl of chloroform according to the manufacturer's instructions (Invitrogen). RNA transcript was precipitated by adding an equal volume of isopropanol and pelleted by centrifugation at 12,000 g for 15 min at 4°C. The RNA pellet was washed once with 75% ethanol and air-dried for 10 min. The dried RNA pellet was dissolved in 8 µl of RNase-free distilled water and treated with 10 U of DNase I for 30 min at 37°C. After inactivating DNase I by heating at 75°C for 5 min, 2 µl of purified RNA transcript was analysed using a one step RT-qPCR method employing the primer set, p21RT-PCRbio (table 1) and the Light Cycler 480 (Roche). The primer set employed in this study produced a single peak in the melting curve analysis, demonstrating that the RT-qPCR reaction produced a single sized amplicon, representing the specific PCR product from the target sequence of PCRp21MLbio (data not shown). Known amounts of template DNA (625 fg to 6,250 fg of PCRp21MLbio) was used to plot the standard curve that was used to calculate the amount of RNA transcript from the *in vitro* transcription reaction (Figure 4C). Control reactions without NTP in the transcription reaction were used to estimate the amount of template DNA carryover in the final RNA preparation. The results from control reactions indicate that less than 0.001% of input DNA template survived from the DNA template depletion procedure (Figure 4D). Three tested conditions, in the presence and absence of p53 and/or p300 produced high basal transcription activities from the *p21* promoter, which reached to more than 150 pg equivalent of the target sequence (Figure 4D). This result demonstrates that the DNA template depletion procedure can successfully suppress the signal from the template DNA in the RT-qPCR assays and can be used to accurately quantify RNA production from the *in vitro* transcription reaction.

### Real time PCR analysis of in vitro transcription assay from a chromatin template

To examine if the RT-qPCR assay can quantify inducible activation of a chromatin-assembled *p21* promoter, 1 µg of PCRp21MLbio DNA was assembled into chromatin according to the procedures described previously [12]. The quality of chromatin assembly was assessed by MNase digestion and showed comparable DNA ladders to the supercoiled plasmid DNA (Figure 4E). Chromatin-assembled PCRp21MLbio was subjected to *in vitro* transcription analysis as summarised in Figure 4A. Briefly, 50 µl of preblocked streptavidin-coupled agarose resin was added to the chromatin assembly reaction containing 1 µg of PCRp21MLbio and incubated at room temperature for 1 hr with constant shaking. The chromatin-immobilized resin was thoroughly washed with HEG50 buffer and finally resuspended in the same wash buffer to yield a final concentration of 16 ng DNA/µl. Then 5 µl of chromatin-immobilized agarose was used for an *in vitro* transcription reaction and subjected to DNA template depletion procedure as described earlier for the *in vitro* transcription of the naked DNA template (Figure 4B). The results show that chromatin assembly on the *p21* promoter, using chromatin-assembled PCRp21MLbio, significantly repressed basal transcription activity. The level of basal RNA production in the absence of p53 and p300 was about one pg whereas naked template in the same conditions produced approximately 150 pg of RNA (compare Figure 4D and Figure 4F). Transcription assays with p53 alone produced a modest enhancement of transcription that was further enhanced by co-incubating with p300 (Figure 4F). These results are similar to those observed using the conventional G-less cassette transcription assay with supercoiled plasmid DNA (p208p21ML) in Figure 3C. Overall, the amount of G-less RNA produced from activation of a chromatin-assembled PCRp208p21 DNA was about 10% of what was observed from the naked DNA template. Albeit not significantly affecting a final RNA quantification, we note that the template DNA amplicon was still present in final RNA preparations even after several elimination steps. Given a short amplicon (62 bp) for RT-qPCR in this study, it is plausible that some of fragmented template DNA by DNase I digestion was still present and amplified by the qPCR reaction. The background level could be further reduced by a thorough blocking and washing of streptavidin-coupled resin and by using a longer amplicon in the RT-qPCR reaction.

This study demonstrates that a one-step RT-qPCR method can successfully replace the radioisotope-labelling of RNA and gel electrophoresis procedure in the G-less cassette *in vitro* transcription assay. By eliminating several time-consuming steps, the whole procedure can be completed in one day, from chromatin assembly to the quantification of RNA product in the final transcription reaction. Since the whole procedure is performed in a liquid-based reaction, this methodology could also be easily adapted to a high throughput format, with the ability to accurately quantify even small amounts of RNA transcript.

## Materials and Methods

### Plasmids and reagents

p208p53ML [9], pWWP-luc [16] and all other expression plasmids were obtained from Dr. Roeder at the Rockefeller University. PCR primers, M2 agarose and FLAG peptide were purchased from Sigma. Enzymes for DNA manipulation were from New England Biolabs. High capacity streptavidin-coupled agarose was purchased from Pierce and aliquoted to 50% suspension in the blocking buffer as described in the text. TRIZOL LS and the SuperScript III Platinum SYBR Green One-Step qRT-PCR kit were purchased from Invitrogen. RNase T1 and DNase I were purchased from Roche Diagnostics. All other chemicals and biochemicals were purchased as molecular biology grade reagents.

### Preparation of recombinant proteins and HeLa nuclear extracts

*E. coli* BL21-Codon plus (Stratagene) was used to express FLAG-p53, Histidine-tagged NAP1 and Xenopus core histones. Sf9 insect cells were maintained in Grace's insect medium (Gibco) supplemented with 10% fetal bovine serum and used to express recombinant drosophila ACF complex (Flag-ACF+ISWI) and p300 from baculoviruses. Detailed purification methods were as previously described [12], [13].

### One-step RT-qPCR

Triplicate samples of 2 µl of purified RNA from *in vitro* transcription reactions were analysed by adding 23 µl of a combined reverse transcriptase (RT) and PCR reaction mixture (0.2 µM forward and reverse primers, 12.5 µl of 2× SYBR reaction mixture, 0.5 µl of reverse transcriptase and Taq DNA polymerase mixture). The RT reaction was started by incubating at 50°C for 30 min for cDNA synthesis and followed by conventional qPCR amplification cycles (95°C for 5 sec and 60°C for 30 sec, 40 cycles) in the LS480 real time PCR machine (Roche).
